# Leveraging cross-view geo-localization with ensemble learning and temporal awareness

**DOI:** 10.1371/journal.pone.0283672

**Published:** 2023-03-30

**Authors:** Abdulrahman Ghanem, Ahmed Abdelhay, Noor Eldeen Salah, Ahmed Nour Eldeen, Mohammed Elhenawy, Mahmoud Masoud, Ammar M. Hassan, Abdallah A. Hassan

**Affiliations:** 1 Computer and Systems Engineering Department, Faculty of Engineering, Minia University, Minia, Egypt; 2 Centre for Accident Research and Road Safety-Queensland (CARRS-Q), Queensland University of Technology, Brisbane, Australia; 3 Department of Information Systems & Operations Management, and Interdisciplinary Research Center for Smart Mobility and Logistics, King Fahd University of Petroleum and Minerals, Dhahran, Saudi Arabia; 4 Arab Academy for Science, Technology, and Maritime Transport, South Valley Branch, Aswan, Egypt; TU Wien: Technische Universitat Wien, AUSTRIA

## Abstract

The Global Navigation Satellite System (GNSS) is unreliable in some situations. To mend the poor GNSS signal, an autonomous vehicle can self-localize by matching a ground image against a database of geotagged aerial images. However, this approach has challenges because of the dramatic differences in the viewpoint between aerial and ground views, harsh weather and lighting conditions, and the lack of orientation information in training and deployment environments. In this paper, it is shown that previous models in this area are complementary, not competitive, and that each model solves a different aspect of the problem. There was a need for a holistic approach. An ensemble model is proposed to aggregate the predictions of multiple independently trained state-of-the-art models. Previous state-of-the-art (SOTA) temporal-aware models used heavy-weight network to fuse the temporal information into the query process. The effect of making the query process temporal-aware is explored and exploited by an efficient meta block: naive history. But none of the existing benchmark datasets was suitable for extensive temporal awareness experiments, a new derivative dataset based on the BDD100K dataset is generated. The proposed ensemble model achieves a recall accuracy R@1 (Recall@1: the top most prediction) of 97.74% on the CVUSA dataset and 91.43% on the CVACT dataset (surpassing the current SOTA). The temporal awareness algorithm converges to R@1 of 100% by looking at a few steps back in the trip history.

## Introduction

The current standard localization technique is the global navigation satellite system (GNSS). Although the GNSS accuracy declines in cases where there are few lines of sight (e.g., urban canyons [1]). Using cross-view geo-localization, a vehicle localizes itself by matching street view images against a database of geotagged images captured from aerial platforms (e.g., a satellite [2] or a drone [3]). Cross-view geo-localization is gaining popularity in the scene of autonomous vehicles [2] and robotic navigation [4]: it compensates for a bad GNSS signal-to-noise ratio. And, it’s preferable to other image-based localization techniques (e.g., landmark and ground-to-ground matching) for the ease of covering new areas. Early work [5] on this technique claims that its main challenge is the lack of visual correspondence between aerial and ground views. Later work, while holding the same claim, shows that there are more challenges: geographic scene changes over time [6–10], poor weather and lighting conditions, lack of orientation information [11], and misalignment between aerial and ground images during training. None of the previous models tries to collectively address these five challenges. To build a holistic solution for the problem, an ensemble model is proposed to fuse the predictions of five independently trained models. Each of these models addresses a different challenge.

Moreover, most of the recent work treats the problem as a 1-to-1 image-matching task. This overlooks the fact that these models get deployed in environments where there’s a continuous stream of input, not a single query image. Taking the temporal nature of the problem into account is a must: the experiments in this work show that the recent models can’t differentiate between highly similar query images, and in the case of a moving vehicle, the consecutive images have a high degree of similarity. Some recent models consider the temporal nature of the problem. For instance, *Markov chain Monte Carlo (MCMC)* algorithms are used in [7, 12–14] to predict the current pose and enforce temporal consistency. In [15], the authors enforce temporal consistency by using a transformer-based trajectory smoothing network. These methods are resource intensive or have strong assumptions about the current state of the vehicle. In this paper, an efficient technique, namely, naive history, is explored to achieve the same goal.

The existing datasets are not suitable for conducting experiments to prove that temporal awareness improves accuracy. The dataset needs to be realistic and collected as a trajectory of close points over a long-running journey to resemble driving in a real environment. CVUSA [16] and CVACT [11] include sparse points on the map. VIGOR [17] includes dense points but doesn’t form trajectories. RobotCar [6] has a limited number of examples. A new derivative dataset based on the BDD100K [18] dataset is introduced to fill this gap.

In summary, the contributions of this paper are:

1. A new ensemble model based on five of the current state-of-the-art cross-view matching networks is introduced. The ensemble model achieves a recall accuracy R@1 of 97.74% on the CVUSA dataset and 91.43% on the CVACT dataset.
2. A new derivative dataset that is suitable for temporal-aware cross-view geo-localization models based on BDD100K is constructed.
3. A meta block: naive history, is developed to make the query process temporal aware. While showing that taking journey history into account minimizes the search space. This reduction in search space improves the accuracy. The accuracy converges to ∼100% with a three-step lookback into trip history on our proposed dataset. This meta block is usable with any cross-view model.

The rest of the paper is structured as follows: The next section is an overview of the related works. Then the proposed ensemble model has been explained followed by the experimental results. In the last section, the conclusion and future work have been presented.

## Related work

The first subsection, starts by investigating how different models engineered their features and architectures. Their choices show how different models tried to approach the problem from different angles, followed by giving a bird-eye-view of the architectures of these models and feature extraction methods. The second subsection walks through the models that tried blending the trip history into the image-matching task. By grouping the models into two categories: one that relies on “this place looks familiar”, and another one that relies on “where have we been before getting here?”.

### Features and architectures

Most of the recent work treated the cross-view geo-localization task as an image retrieval task. They tried to find a feature representation suitable for matching query ground images and aerial ground images. The nature and complexity of used networks changed over time. Here, a brief comparison is conducted between the most common feature representations and their architectures. Table 1 summarizes the feature types and the backbones of different cross-view geo-localization networks.

**Table 1:** A summary of the feature types and the backbones of different cross-view geo-localization networks.

Feature type	Backbone	Used in
Hand-crafted	SIFT	[12, 19]
Hand-crafted	SURF, FREAK, PHOW	[12]
Hand-crafted	SIFT + VLAD	[7]
Semantic	Faster R-CNN	[20]
CNN	VGG + FCN + NetVLAD	[14, 21, 22]
CNN	AlexNet	[5, 20]
CNN	VGG	[11, 13, 23]
CNN	ResNet	[11, 13, 24]
CNN	DenseNet	[11, 13]
CNN	U-net	[11]
CNN	Xception	[13]
CNN	-	[25]
CNN	VGG + FCN	[26]
Attentive	Siam-FCANet (ResNet + FCAM + FCN)	[27]
Attentive	Siam-VFCNet (ResNet + FCAM + NetVLAD)	[27]
Attentive	VGG + SAFA + SPE	[28–30]
Attentive	VGG + Geo Attention + Geo-temporal Attention	[15]
Attentive	ResNet + Self Cross Attention	[8]
Attentive	SAFA	[17]
Attentive	ResNet + SAFA	[31]
Attentive	ResNet + Self Cross Attention	[32]
Attentive	VGG + MSAE	[33]
Synthesized	X-Fork	[34]

#### Hand-crafted features

Early research used hand-crafted features. SIFT [35] was used in [12, 19]. The authors of [12] experimented with other feature spaces (e.g., SURF [36], FREAK [37], PHOW [38]) but SIFT outperformed others. Dense SIFT features were computed in [7], then embedded into a higher dimensional vector space using a VLAD [39]. The extracted features in this category were brittle; it failed to adapt to appearance change. Later, it proved to have inferior performance compared to the CNN-based features.

#### Semantic features

In this approach, the networks matched ground and aerial images based on the meaningful content of the image. This made it more robust to viewpoint changes than local features. But the model performance degraded in areas lacking the pre-selected semantic features. In [20], the authors treated the problem as object detection and recognition: the first block of the architecture employed the Faster R-CNN [40] to detect buildings, and the second block used AlexNet [41] with the Siamese architecture [42] to recognize the buildings.

#### CNN-based features

Metric learning achieved promising results in bridging the domain gap between aerial and ground image representation. In [21], the authors used a fully convolutional network (FCN) with a NetVLAD [41] layer using a Siamese architecture. The authors of [5] tried modified versions of AlexNet. The authors of [13] experimented with different FCN layers: VGG [39], ResNet [43], DenseNet [44], and Xception [45] with the same architecture of [21], they found that VGG outperforms other networks. In [14], the authors used the CVM-Net-I architecture proposed in [21]. In [24], the authors exploited a modified ResNet50 network for ground images and a ResNet18 for aerial ones. The authors of [23] used VGG16 to generate the feature maps for the polar transformed aerial and then fed it into the Dynamic Similarity Module (DSM). In [11], the model learned orientation information by using different backbones (VGG, ResNet, DenseNet, and U-net [46]) with the Siamese architecture. The authors of [26] employed the Siamese network with a VGG backbone to extract feature maps, then a fully connected layer aggregates these feature maps. In [25], the authors used a CNN to generate feature maps and then transform them from the ground domain to the aerial domain. The authors of [22] applied the hybrid perspective mapping method using the CVM-Net-I architecture.

#### Attentive features

For this feature type, the networks used spatial attention to enhance the feature representation. In [27], the authors integrated the lightweight attention module (FCAM) into each block of the basic ResNet. The authors of [28] used a spatial-aware feature aggregation (SAFA) module to mitigate the distortion in the aerial image, then employed the spatial-aware position embedding module (SPE) to encode relative positions among features in the feature maps. The authors of [17] proposed the VIGOR network built on top of SAFA. In [29, 30], the authors used the same architecture as [28] with a different loss function: *geo-distance weighted loss*. In [15], the authors proposed a geo-attention module for the aerial branch and a temporal-attention module for the ground branch. The authors of [8] used convolutional block attention modules [47] to generate multi-scale attention features. The model built in [31] employed a modified ResNet34 backbone with a spatial-aware attention module. In [32], the authors proposed the EgoTR network. EgoTR used a ResNet backbone transformer encoder with a self-cross attention mechanism. The authors of [33] introduced the Multi-Scale Attention Encoder (MSAE). MSAE employed a VGG backbone with a multi-scale attention encoder followed by FCN to generate feature masks.

#### Synthetic features

In this approach, the networks learned robust feature representation by reversing the task: it learned how to create ground views from aerial views, which made it learn salient features and suppress others. The model built in [34] synthesized aerial representation of a ground panorama query using the X-Fork network [48] with edge maps detection by Canny Edge Detection [49].

### Contextual awareness

The fact that these models get deployed in autonomous vehicles, makes it obvious that the models should be aware of the trip context. Contextual awareness can be categorized as follows:

#### Spatial awareness

We have to differentiate between two types of spatial awareness.

- Some models refer to it as the knowledge about the pose (location and orientation) of the query image and its relative pose to the aerial image frame. In [29, 30], the authors constructed mini-batches of images within a certain geographic radius and used a modified version of the triplet loss function: *Geo-distance weighted loss* to favor examples where the images are within the selected radius. The Geo-Attention module used in [15] exploited a similar loss function. The authors of [22] employed hybrid perspective mapping to establish correspondence between ground and aerial images. The model built in [11] injected the orientation information into the network and used multiplane image (MPI) [23] projections to exploit geometric correspondence between ground and aerial images. Table 2 gives an overview of spatial (pose-wise) awareness approaches used in cross-view geo-localization.
- Other networks refer to it as paying more attention to the salient features, suppressing less important features, and encoding the spatial layout information into the feature representation.

**Table 2:** An overview of the spatial (pose-wise) awareness approaches used in cross-view geo-localization.

Approach	Used in
Geographic proximity	[15, 29, 30]
Polar Transform	[3, 11, 17, 22, 23, 30–32]
Inverse Polar Transform	[33]
Orientation	[11]
Dynamic Similarity Matching (DSM)	[23]
Hybrid Perspective Mapping	[22]

#### Temporal awareness

There are two types of temporal awareness too!

- Finding a robust feature representation that won’t be affected by scene changes throughout time. These changes can be geographic landmarks (e.g., new constructions) or environmental conditions such as weather conditions. In [6, 9], the authors used time-invariant approaches by capturing the same scene during different conditions across time. But, this technique required a dataset where the same scene is covered during different conditions which are challenging to collect. Possible solutions for this are as follows: **A)** Authors of [8] used semantic object-based data augmentation techniques to remove and add objects (cars, roads, greenery, and sky). **B)** Authors of [7] applied PCA projection to make background features less significant in the image descriptor. **C)** Another solution is using synthetic datasets.
- Exploiting the fact that these models will be deployed on vehicles where a temporally coherent sequence of images is available. Authors of [7, 12–14] used MCMC algorithms, namely, a particle filter [27] with variations of initialization and transition techniques, the algorithms are used to predict current pose and enforce temporal consistency. In [15], the authors introduced a geo-temporal attention module, the module attends to all frames in a video of ground footage to learn better features, also, it enforces temporal consistency by using a transformer-based trajectory smoothing network.

Table 3 gives an overview of the temporal awareness approaches used in cross-view geo-localization.

**Table 3:** An overview of the temporal awareness approaches used in cross-view geo-localization.

Approach	Used in
Capture multiple examples with different environmental conditions	[6, 9]
Semantic object-based data augmentation	[8]
Observation encoder + PCA projection	[7]
MCMC algorithms	[7, 12–14]
and Sequence attention + Trajectory Smoothing Network	[15]

The majority of the works treat the problem as a 1-to-1 image-matching task, ignoring the reality that these models are deployed in environments with a continuous stream of data rather than a single query image. The models that include the temporal character of the problem are resource costly or make heavy assumptions about the vehicle’s current condition.

## Methodology

### Evaluation metric

In this research, the same evaluation metric used in [3, 8, 11–15, 17, 21–23, 25–34] is used: the **Recall**@*k* (**r**@*k*). For r@*k*, it’s considered a match if the corresponding aerial image is in the top *k* predictions. r@1, r@5, r@10, and r@1% are used. r@1 means the true image is the first prediction of the model, r@5 means the true image is in the first five predictions of the model, and so on.

### The ensemble model

There are many challenges in cross-view matching. To name a few: missing correspondence and orientation information, scene changes over time, and the high similarity among geographically close points. Recently, several models were developed to solve the cross-view (CV) matching problem, and different models approached the CV matching problem from different angles. Thus, each model has its advantages and disadvantages. In this research, it is hypothesized that aggregating the outputs of these uncorrelated models might improve the accuracy. So, in this research, an ensemble model of independently trained models is built to solve the CV matching problem. The proposed ensemble uses the same datasets to train different neural network architectures. The CVUSA [16] and CVACT [11] benchmark datasets are used to train five models. Each model addresses a different aspect of the problem: DSM [23] estimates the cross-view orientation alignment. EgoTR [32] models global dependencies to decrease visual ambiguities and matches geometric configuration between ground and aerial images. SAFA [28] exploits geometric correspondence between aerial and panoramic ground images. Toker [31] biases the localization network via a Generative Adversarial Network (GAN) to learn salient features. SFCANet [27] uses Hard Exemplar Reweighting to assign a greater weight to hard examples. Table 4 shows their r@*k* metrics.

**Table 4:** The r@*k* metrics for the networks used to construct the ensemble model.

Network	CVUSA				%	CVACT			
	r@1	r@5	r@10	r@1%		r@1	r@5	r@10	r@1%
DSM [23]	92.07	97.33	98.33	99.60		81.93	91.83	93.95	97.42
Toker [31]	92.15	97.28	98.26	99.56		83.52	93.89	95.48	98.17
EgoTR [32]	93.87	98.22	98.98	99.66		84.87	94.5	95.95	98.37
SFCANet [27]	51.01	78.92	86.80	98.49		x	x	x	x
SAFA [28]	88.93	96.65	97.97	99.65		74.56	89.65	92.46	97.72

**Fig 1:**
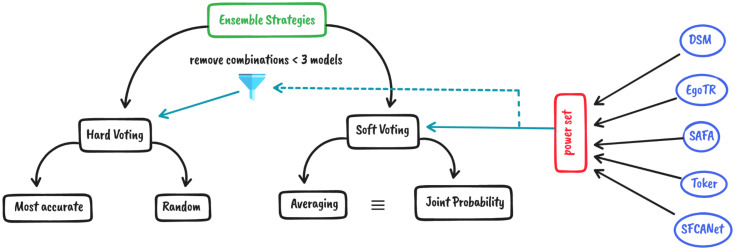
Different ensemble aggregation methods.

This research investigates two different aggregation methods: soft-voting, and hard-voting. As shown in Fig 1, for both methods, all possible combinations of the models (their power set) are tried. Five state-of-the-art models are used and their outputs are combined using 32 different combinations for each strategy.

In soft-voting, two calculation methods are tried: **A)** averaging the predictions of the individual models. **B)** calculating the joint probability, using Eq (1). Both methods have identical results.

$$\begin{array}{c} \text{total}\;\text{vote}=\text{exp}(\text{log}\left(vote_{1}\right)+\text{log}\left(vote_{2}\right)+\ldots ,\text{log}\left(vote_{n}\right)), \end{array}$$ (1)

In hard-voting, the majority vote of the models is selected. Hard-voting needs at least three models to have a majority vote. There are two cases where a majority vote doesn’t exist:

1. Combinations with an even number of models can tie, the combination containing the most accurate model is picked.
2. All models’ predictions differ, two strategies are tried: **A)** Take the prediction of the most accurate model in the combination. **B)** Pick a prediction from a random normal distribution of individual models’ predictions.

### BDD trajectories dataset collection

The proposed naive history meta block exploits the temporal nature of the problem. The existing benchmark datasets (e.g, CVUSA, and CVACT) are collected from sparse points on the map while a trajectory of close points is needed. Inspired by the work of Regmi and Shah [15], A new derivative dataset from the BDD100k [18] dataset is constructed to address this gap. The BDD100K dataset is crowdsourced, diverse, and large-scale, with IMU/GPS recording, and other semantic annotations (irrelevant to this research). All videos in the dataset are 40*s* long, though the total distance varies. The videos with a distance greater than 50 are chosen, the statistical summary of the distance covered in the selected videos is in Table 5.

**Table 5:** A statistical summary of the distance covered in selected videos.

Count	Mean	*std*	Min	Max
47943 videos	278.668 m	181.786 m	50.000 m	2560.000 m

**Fig 2:**
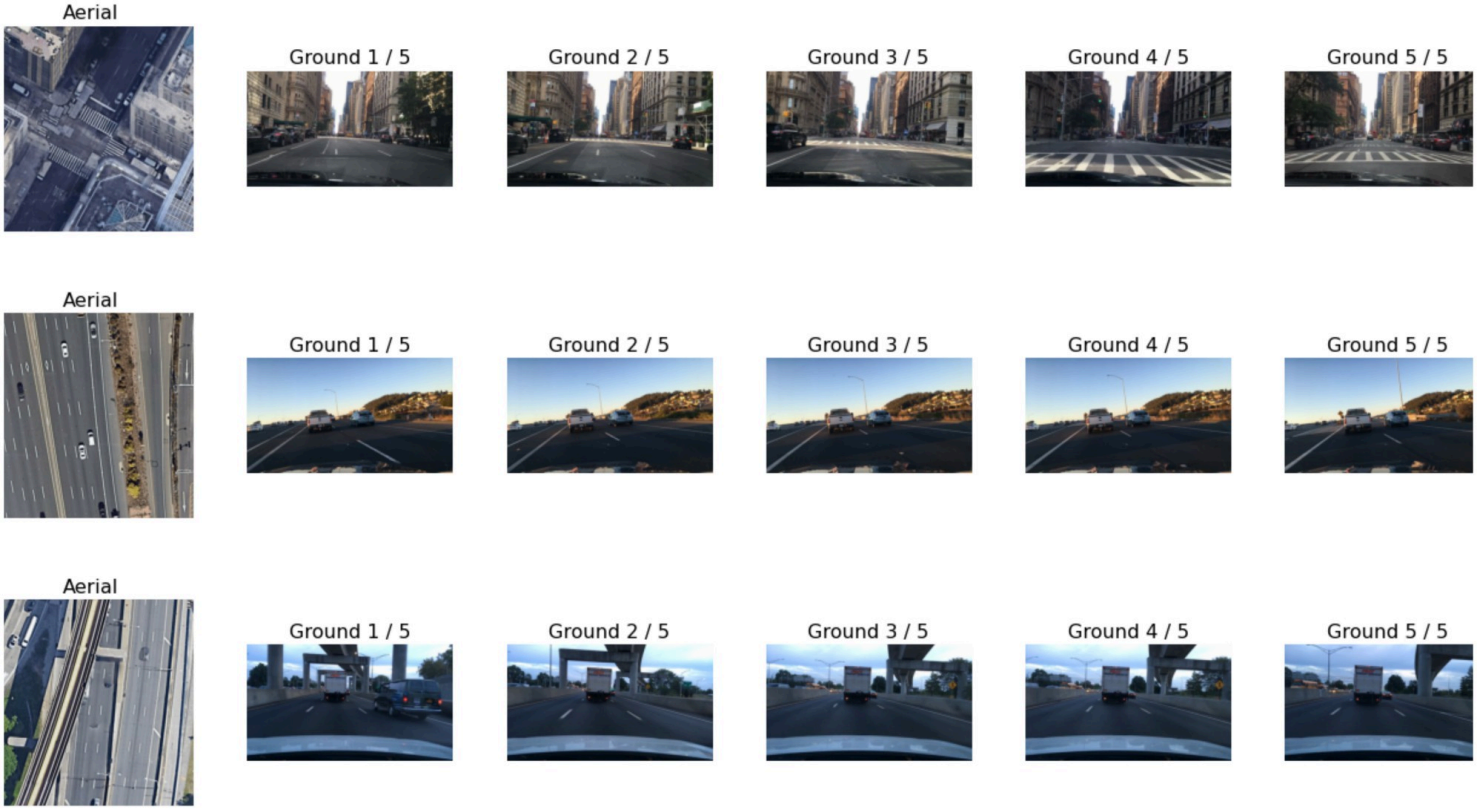
Three examples from our dataset for ground images.

The proposed dataset consists of 95,000 examples. Each example consists of five ground images and one aerial image, and some examples are shown in Fig 2. Note that in the figure, time progresses from left to right and the distance between the consecutive frames is 10 m. The ground images are sampled from the picked videos with a sampling rate of 1/*frame*/10*m* to have some visual changes between the consecutive frames, but at the same time, the frames still look relatable to one another. The *IMU/GPS* data is captured at 1 *sample*/*s*. The distance moved in one second varies; the speed of the vehicles is not constant. The proposed dataset cares about the visual changes, not the passage of time. So when the distance between every two consecutive locations is greater than 10 *m*, the following sampling algorithm is used to sample uniform trajectories:

**Algorithm 1**: BDD100K resampling

**Data**: The GPS/IMU information recorded along with the videos, BDD100K videos.

**Result**: Resampled frames(1 frame/10 m)

/* Get the speed at the current (*v*_*n*_), and next (*v*_*n*+1_) location from IMU data. Assume the speed between these two locations (*v*_*n*→*n*+1_) is the average speed of both locations. For all locations on the trajectory which is a multiple of the sampling distance parameter (*d*):       */

**1** $v_{\text{n}\to \text{n+1}}\leftarrow \frac{v_{\text{n}}+v_{\text{n}+1}}{2},n\in \{1,2,3,\ldots ,\left\lfloor \frac{\text{trajectory}\;\text{distance}}{d}\right\}$;

/* To get the timestamp of the nth frame (*t*_n_)      */

**2** $t\leftarrow \frac{\text{d}\times \text{n}}{v_{\text{n}}+v_{\text{n}+1}},n\in \{1,2,3,\ldots ,\left\lfloor \frac{\text{trajectory}\;\text{distance}}{d}\right\}$;

**3** Extract the frames at the selected timestamps using FFmpeg [50];

The aerial tiles are captured at the midpoint of the example using the great circle algorithm [51] and then fetched from Google Maps [52]. Different zoom levels are experimented. The 20th zoom level is chosen; it covers a wide area with great detail. A tile size of 800 × 800 is chosen.

**Fig 3:**
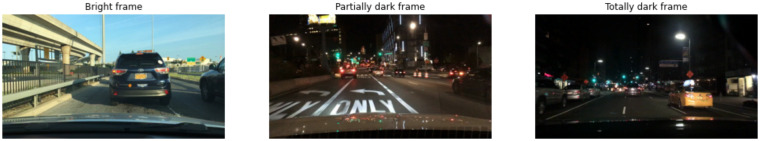
Examples of different lighting conditions in the BDD100K dataset.

**Fig 4:**
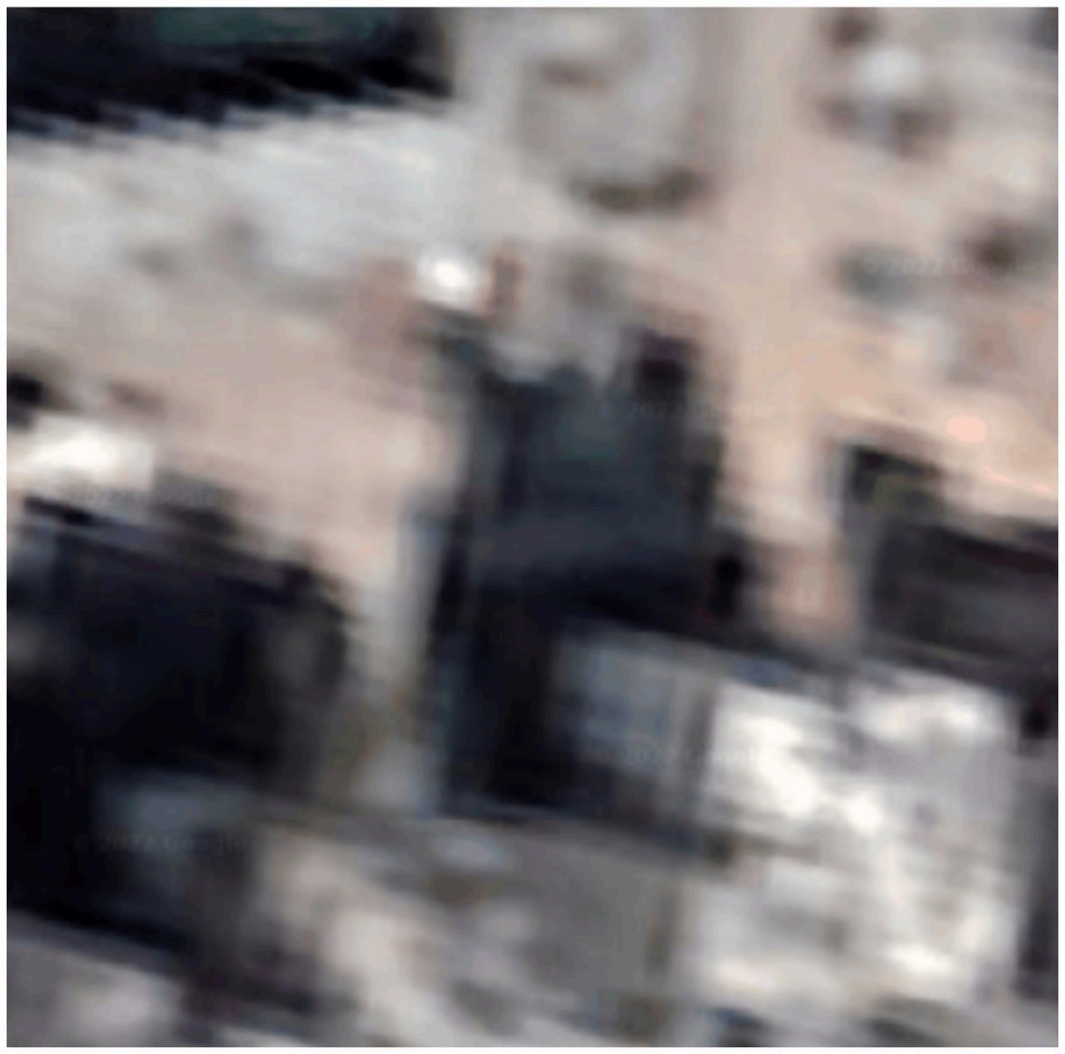
An example of a blurry aerial image. Sometimes this is deliberate by the satellite imagery provider for privacy reasons.

**Fig 5:**
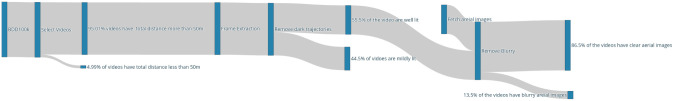
A simplified version of the data processing pipeline.

After extracting the frames, trajectories where 30% of the extracted ground frames are mildly lit (44.5% of all trajectories) are removed. For example, the trajectory of the Bright frame in Fig 3 is accepted and the other two trajectories are dropped. In the dark frames, no meaningful correspondence between the ground and aerial images can be constructed. Then the trajectories that have blurry aerial tiles (13.5% of the bright trajectories) are removed (similar to the example shown in Fig 4). A Laplacian filter [53] with a threshold of 200 is used to detect blurry aerial tiles, and a gray scale mean filter with a threshold of 85 is employed to detect dark ground frames. Both thresholds are chosen empirically to drop all the true positive corrupt examples with some false positives. Fig 5 shows a simplified dataflow of the data processing pipeline. This dataflow pipeline is *embarrassingly parallel*. It successfully ran across a 6-machines-cluster, with 16 cores each. After that, the ground and aerial images’ width is scaled to 400px while keeping the height in aspect ratio using the *Lanczos algorithm*.

In contrary to the GAMa dataset [54], the examples in the BDD-trajectories dataset are evenly-spaced and the examples where the crosspondence between the ground and aerial images is imperceptible (due to lighting conditions or satellite capture policies) are removed.

### Naive history

Inspired by the experiments on the joint probability as a soft-voting strategy for ensemble learning. In a journey setting where the history of the journey is available, it is hypothesized that taking the previous predictions into account might cause the prediction to converge while progressing in the journey. This hypothesis relies on the fact that even if the model doesn’t return the true location as its top-1 prediction, most of the time, it is still in the top-1% predictions. Also, the probability that a model will predict *N* consecutive wrong predictions decreases as *N* increases. Based on this, the EgoTR model is fine-tuned on the new derivative dataset (*BDD-trajectories*). The BDD-trajectories dataset is used because it has trip trajectories.

#### Fine-tuning EgoTR

The *EgoTR* model takes a pair of images as input: a ground image and a satellite image as its input. However, an example in the proposed dataset consists of a hexad: five ground images and a satellite image. The proposed dataset has to get reshaped to be suitable for the EgoTR input format. The chosen examples are the ones where there are at least two examples from the same journey, that is 10 ground images from the same trajectory. Every ground image gets paired with the satellite image from the example. This means that each example in *BDD-trajectories* corresponds to five examples in the reshaped dataset. A subset of reshaped dataset is used: 19015 pairs for validation and 75985 pairs for fine-tuning.

#### Attaching the naive history block to EgoTR

After fine-tuning EgoTR, the distance array is generated for all the images in the proposed validation dataset. Then this array is fed as an input for Algorithm 2.

**Algorithm 2**: Naive history

**Data**: *distanceArray*_*ij*_, *historyDepth* ≥ 1

**Results**: History reinforced distance array

**1** *m*_*ij*_ ← *distanceArray*_*ij*_;

**2** *D* ← *historyDepth*;

**3** *len* ← |*m*_*ij*_|;

**4 for** *historyDepth* ∈ [1, *D*] **do**

**5**  *prevDistanceArrayLen* ← *len* − *historyDepth*;

**6**  $prevStepDistanceArray\leftarrow {\left(m_{ij}\right)}_{\begin{array}{c} 1\leq i<prevDistanceArrayLen\\ 1\leq j<prevDistanceArrayLen \end{array}}$;

**7**  $partialHistory\leftarrow {\left(m_{ij}\right)}_{\begin{array}{c} D\leq i\\ D\leq j \end{array}}$;

**8**  *historyReinforcedDistanceArray* ← *prevStepDistanceArray* ⊙ *partialHistory*;

**9**  ${\left(m_{ij}\right)}_{\begin{array}{c} D\leq i\\ D\leq j \end{array}}\leftarrow historyReinforcedDistanceArray$;

**10 end**

**11 return** *m*_*ij*_;

The *naive history* meta block reinforces the prediction at the current position by looking back into the trip history. The *distanceArray* parameter is the distance between every ground and aerial image (the output of the model). The *historyDepth* parameter controls how deep the algorithm looks back into history. The total distance array is shifted by *historyDepth* columns and rows to get the distance array at the previous location (Algorithm 2, Line 6). A shifted version of the *distanceArray* is kept to leave the predictions at future steps intact (Algorithm 2, Line 7). The distance array at the current step and the distance array at the previous step are multiplied, element-wise (⊙) (Algorithm 2, Line 8). The original array gets updated with the reinforced predictions (Algorithm 2, Line 9). Repeat the steps 4 through 7 (Algorithm 2, Line 4), with increasing value of *historyDepth* until reaching the value of final history depth value, for example, if the algorithm wants to look back five steps in trip history it will iterate over *historyDepth* {1, 2, 3, 4, 5}.

#### The effect of prior on naive history

As mentioned earlier, the cross-view models are complementary to existing GNSS, so the naive history performance can be improved by initializing it with a weak prior (the location captured by the GNSS). Algorithm 3 initializes the distance array (generated by the model) with a probability of the first image in each example equal to 1e−6. In other words, in (Algorithm 3, Lines 5-9) the probability of the first image in the trajectory is modified to make the probability of the ground truth image equal 1e-6 and set other probabilities to a uniform value of (1 − (1e−6)/*distancearraysize*). That experiment proves that initializing the naive history algorithm with this prior knowledge speeds up the convergence to 100% accuracy significantly.

**Algorithm 3**: Naive history with a weak prior

**Data**: *distanceArray*_*ij*_, *historyDepth* ≥ 1, *priori* ∈ *R*_+_, *trajectorySize* ∈ *N*

**Result**: Prior-aware history reinforced distance array

**1** *m*_*ij*_ ← *distanceArray*_*ij*_;

**2** *D* ← *historyDepth*;

**3** *len* ← |*m*_*ij*_|;

**4** *normalizer* ← *priori*/*len*;

**5 for** *k* ∈ [0, *len*] ∧ *k* % *trajectorySize* ≡ 0 **do**

**6**  $currentPrediction_{ij}\leftarrow {\left(m_{ij}\right)}_{\begin{array}{c} i\\ j\equiv k \end{array}}$;

**7**  (*currentPrediction*_*ij*_)_*i*≡*k*_ ← *priori*;

**8**  ${\left(m_{ij}\right)}_{\begin{array}{c} i\\ j\equiv k \end{array}}\leftarrow currentPrediction_{ij}-normalizer$;

**9 end**

**10 for** *historyDepth* ∈ [1, *D*] **do**

**11**  *prevDistanceArrayLen* ← *len* − *historyDepth*;

**12**  $prevStepDistanceArray\leftarrow {\left(m_{ij}\right)}_{\begin{array}{c} 1\leq i<prevDistanceArrayLen\\ 1\leq j<prevDistanceArrayLen \end{array}}$;

**13**  $partialHistory\leftarrow {\left(m_{ij}\right)}_{\begin{array}{c} D\leq i\\ D\leq j \end{array}}$;

**14**  *historyReinforcedDistanceArray* ← *prevStepDistanceArray* ⊙ *partialHistory*;

**15**  ${\left(m_{ij}\right)}_{\begin{array}{c} D\leq i\\ D\leq j \end{array}}\leftarrow historyReinforcedDistanceArray$;

**16 end**

**17 return** *m*_*ij*_;

## Results and discussion

### The ensemble model

Although there are multiple options for building the ensemble model (e.g., stacking, boosting, and mixing models). Mixing models (i.e., voting) are chosen for this research for the following reasons:

- If stacking models are used, there will be a need to train the base models alongside the meta model, otherwise, the meta model would overfit to the base models. Base models retraining would require a lot of computational resources.
- All member models in the ensemble are trained on the entire dataset which means the meta learner would also train on the same data as the base model. The other option is to train all the models from scratch, but this would require a lot of training resources.
- Boosting models is not a good option because the base models are independent: the models have different weaknesses and strengths.
- More importantly, the main goal is to show the need for holistic solution for the problem, rather than showing the effectiveness of the ensemble model: in real time scenarios, a single model with something like the naive history algorithm would be much more efficient than an ensemble model.

**Fig 6:**
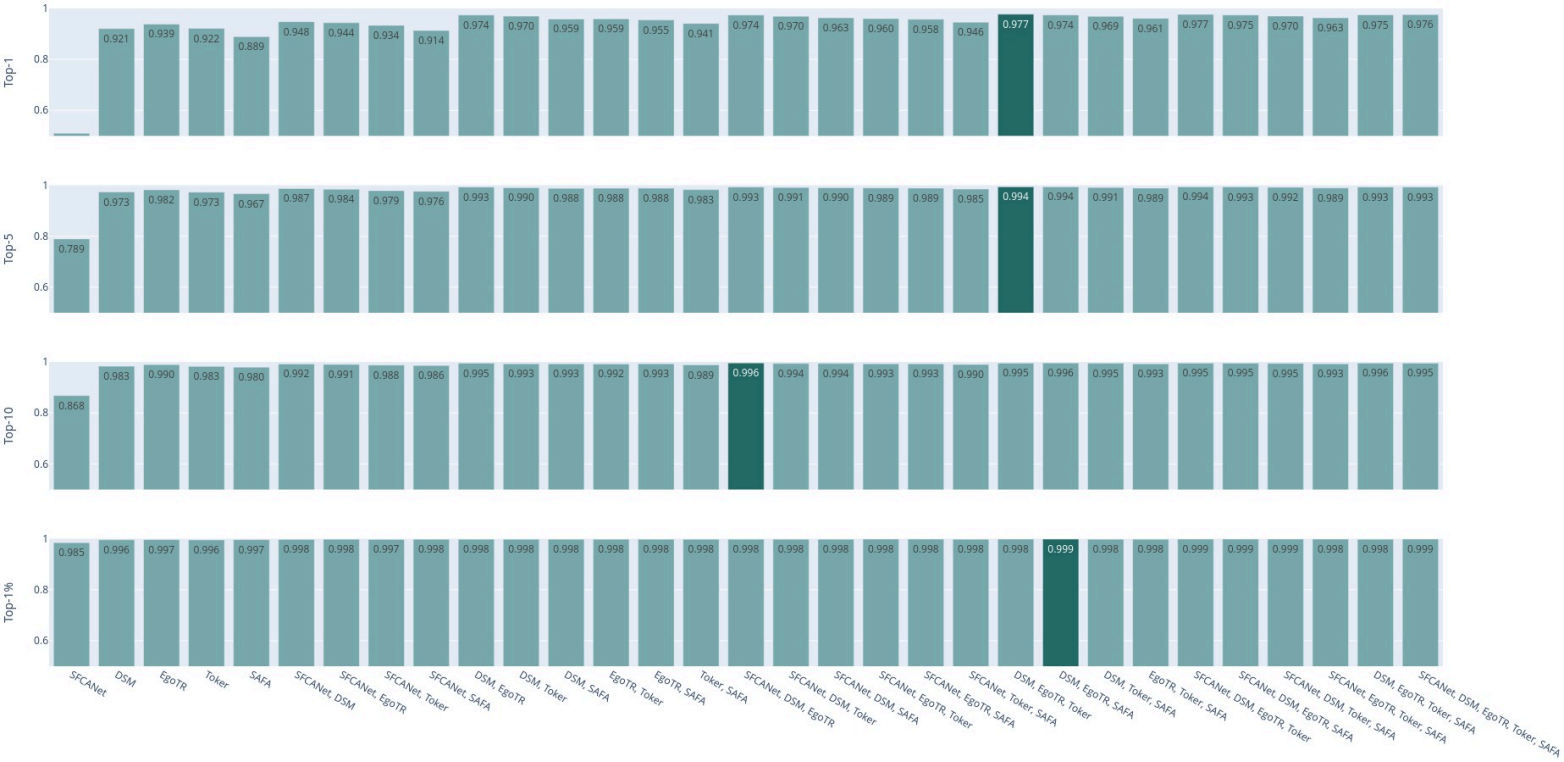
CVUSA combinations for the soft-voting strategies.

**Fig 7:**
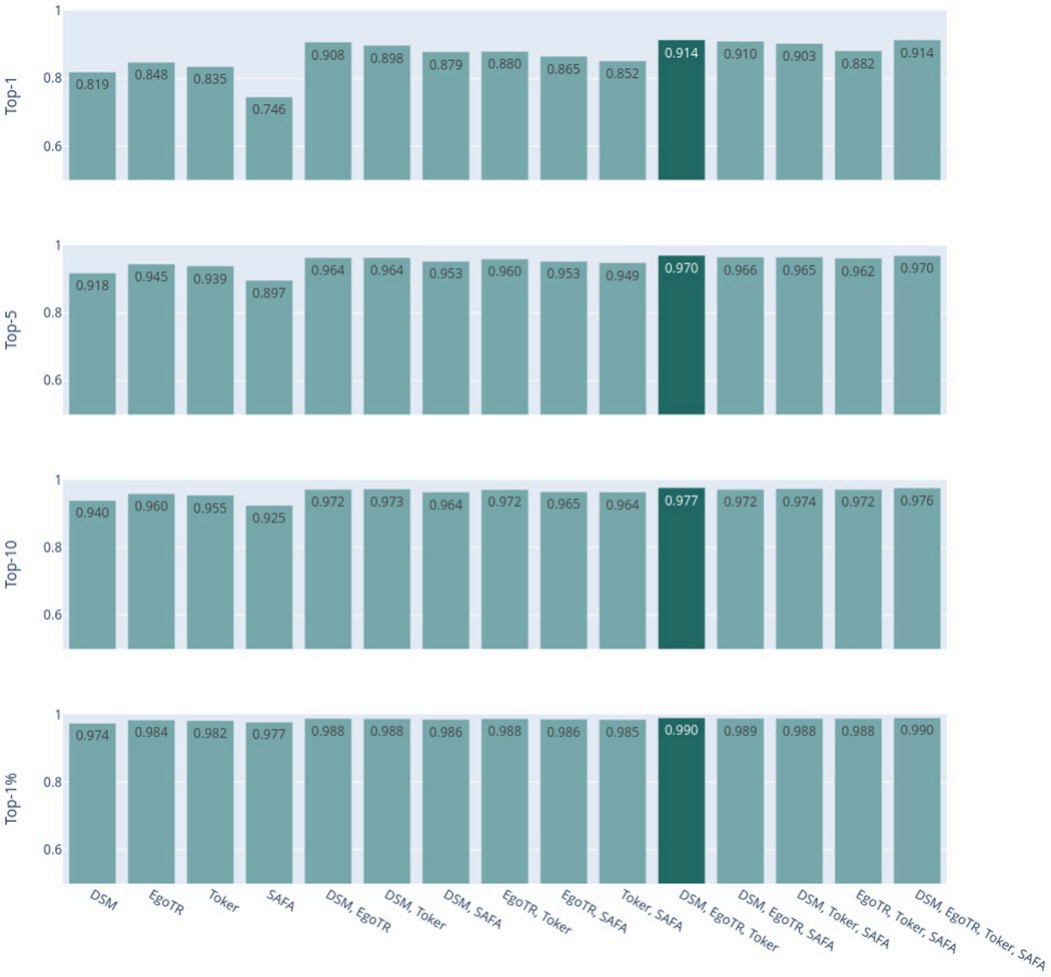
CVACT combinations for the soft-voting strategies.

Now focusing on the voting ensemble model: Figs 6 and 7 show the results of the soft-voting strategies for CVUSA and CVACT, respectively. The DSM, EgoTR, and Toker combination outperforms other combinations (the dark bars in both figures). That is because these three models solve three orthogonal parts of the problem: orientation, geometric correspondence, and coaching the right features. Increasing the number of the models does not necessarily improve the accuracy. To improve the accuracy, individual models have to predict different examples correctly. Although this is mitigated by using a weighted voting strategy.

**Fig 8:**
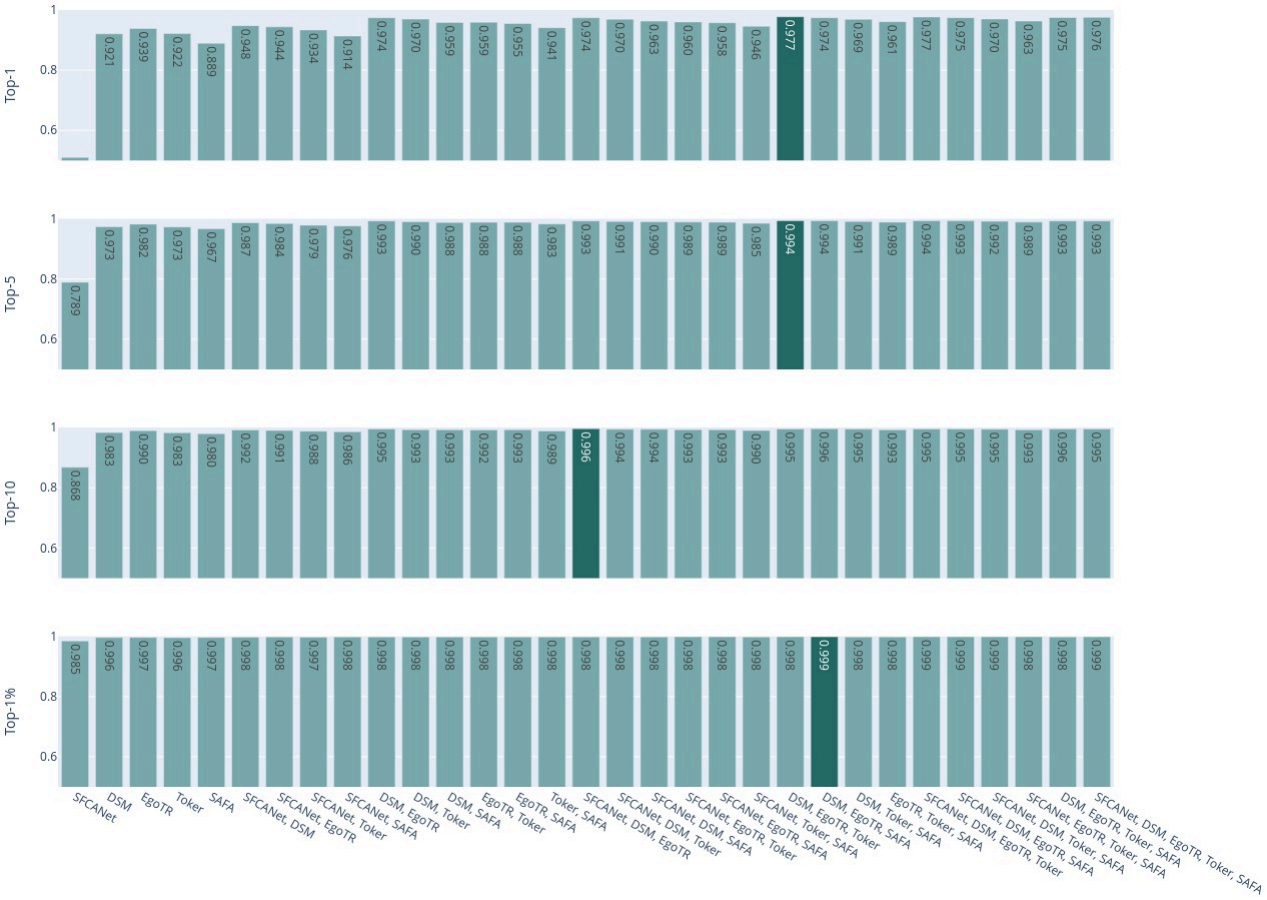
CVUSA combinations for hard-voting with the most accurate model prediction strategy.

**Fig 9:**
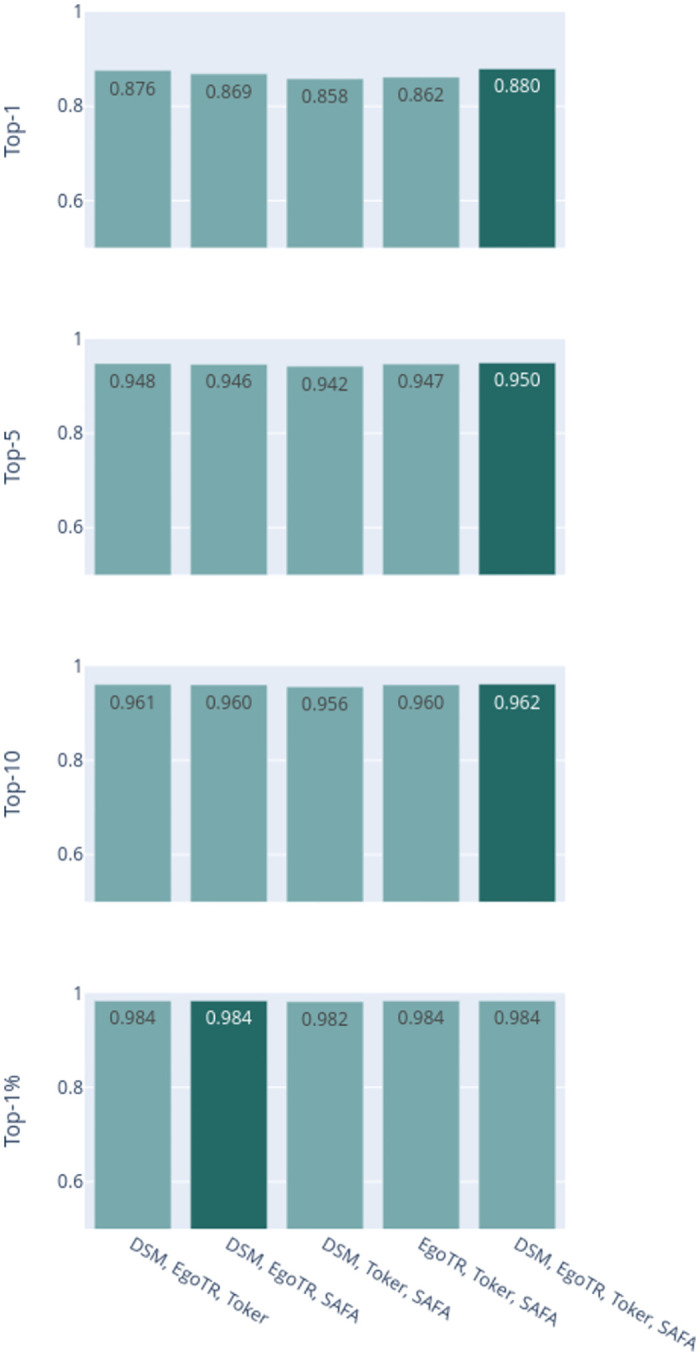
CVACT combinations for hard-voting with the most accurate model prediction strategy.

Figs 8 and 9 show the results of hard-voting with the most accurate model prediction strategy for CVUSA and CVACT, respectively. The DSM, EgoTR, Toker, and SAFA combination outperforms other combinations (the dark bars in both figures). The accuracy drops about 2% for the r@1 and r@5 metrics for CVACT. Rarely all models return the ground truth in the top-5 predictions for CVACT. In some situations the ensemble only have wrong choices to choose from. This was well-compensated by soft voting: the distances calculated by the joint probability can leverage the ground truth prediction even if it isn’t in the top-5. The ensemble didn’t have the same issue with CVUSA because the majority of models return the ground truth in the top-5 prediction.

**Fig 10:**
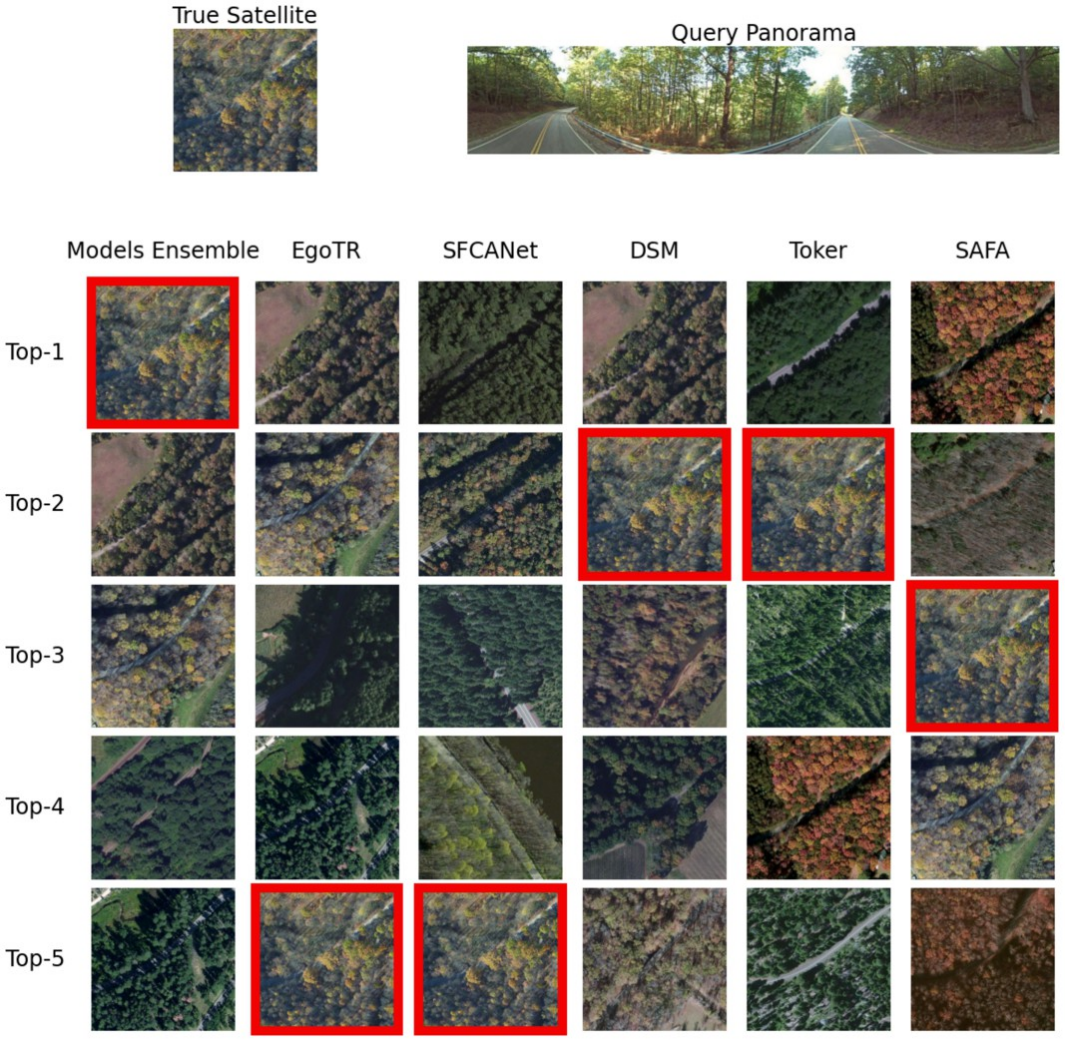
An example that shows the ensemble model r@(1—5) compared to the individual models on the CVUSA dataset. The true satellite has a red border.

**Fig 11:**
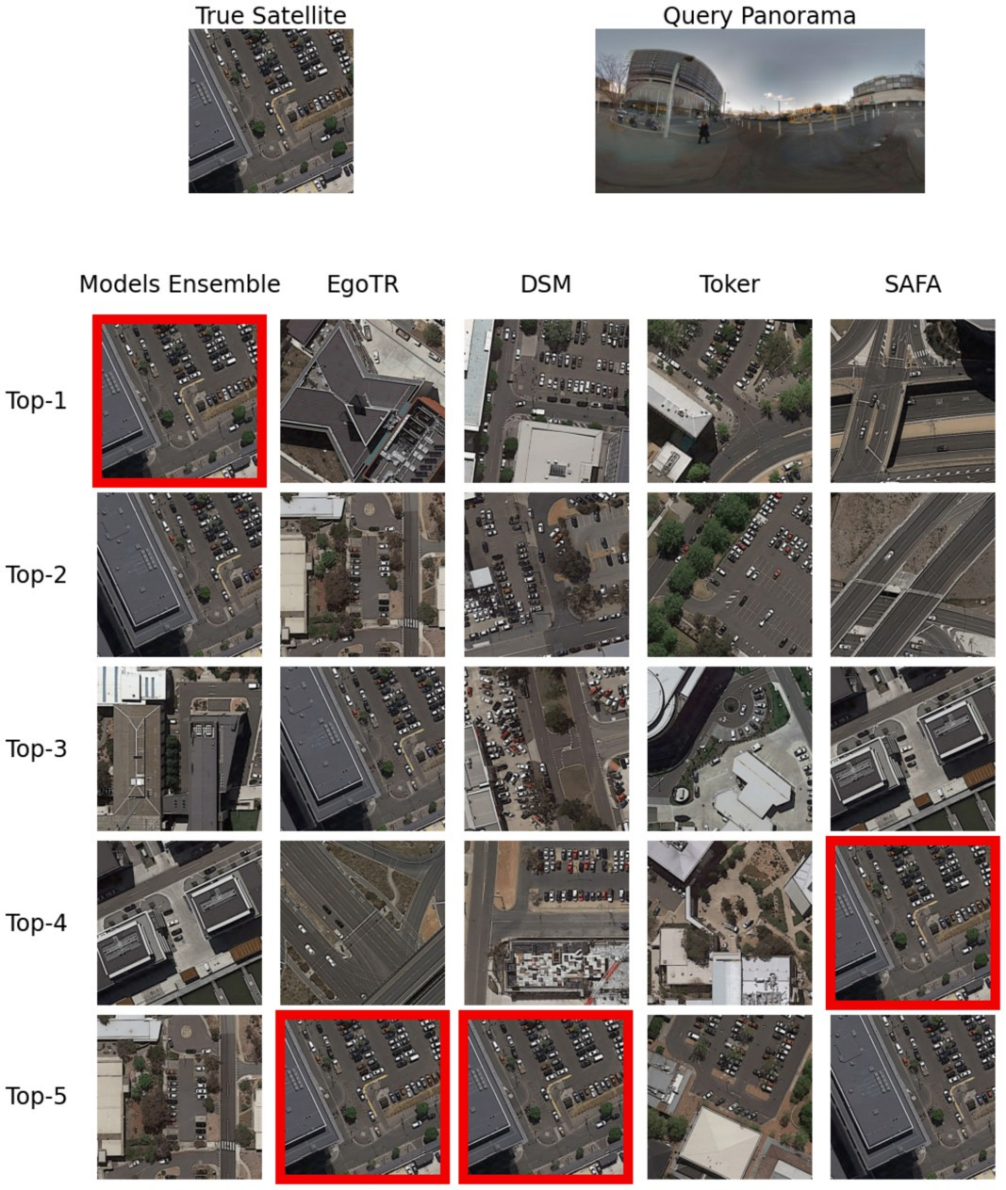
An example that shows the ensemble model r@(1—5) compared to the individual models on the CVACT dataset. The true satellite has a red border.

Soft-voting improves accuracy by looking at the **top-k predictions** across different models. For the sake of illustration, Figs 10 and 11 show an example of the predictions of the individual models. None of the models returned the right prediction as the first prediction, but it was in the top-5 predictions for most models. Their collective prediction (the model ensemble) could return it as the first prediction.

**Fig 12:**
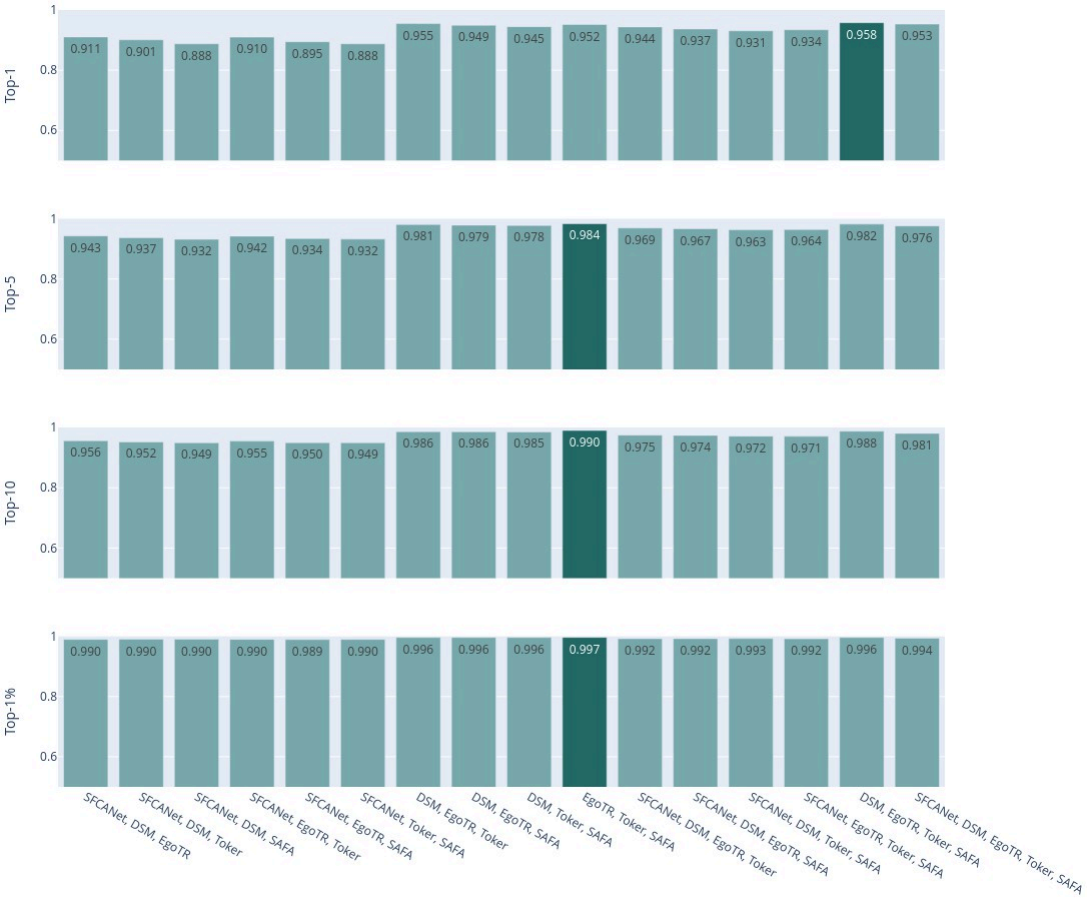
CVUSA combinations for hard-voting with the random selection strategy.

**Fig 13:**
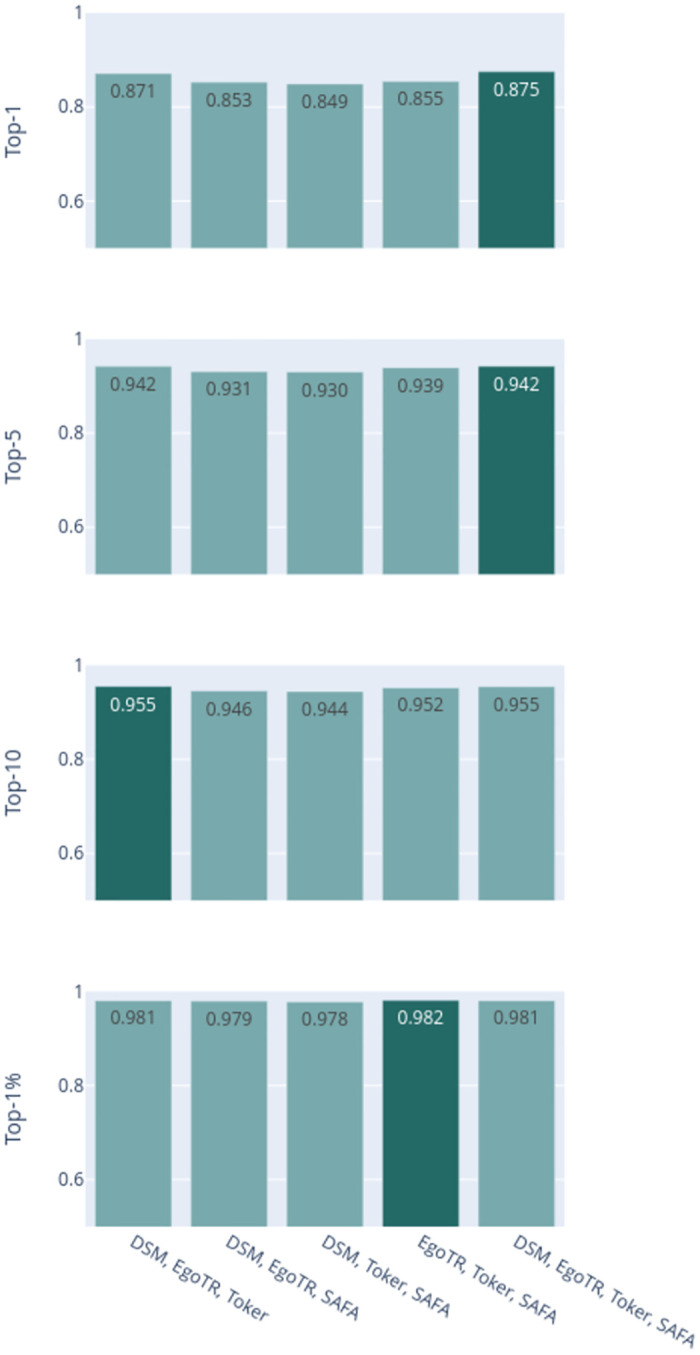
CVACT combinations for hard-voting with the random selection strategy.

Figs 12 and 13 show the results of the hard-voting with the random selection strategy for CVUSA and CVACT, respectively. Here after removing the bias for the most accurate model, increasing the number of voting models improves the result; there is a higher probability of having the right ground truth in the votes pool. The DSM, EgoTR, Toker, and SAFA combination outperforms other combinations (the dark bars in both figures).

**Fig 14:**
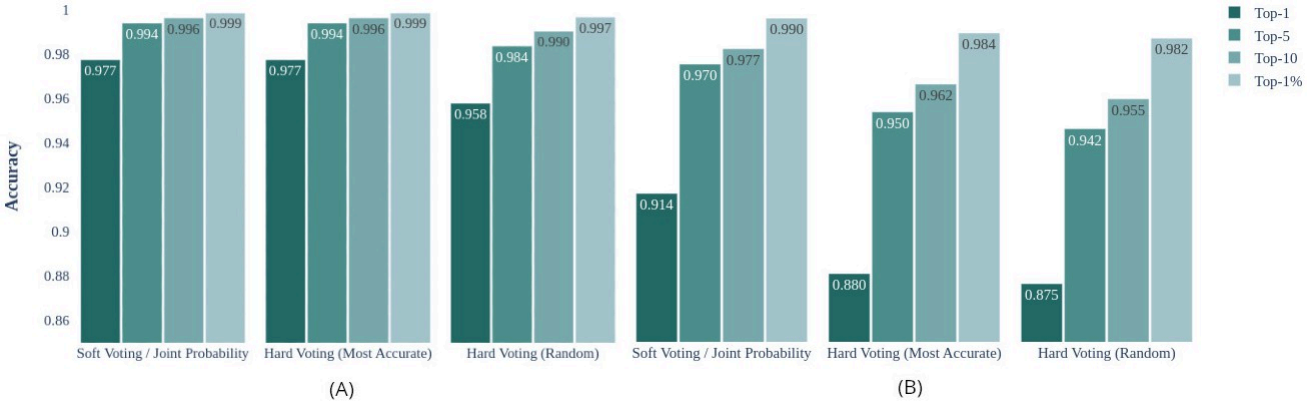
Comparison between aggregation method for the best performing combinations. **A**: CVUSA and **B**: CVACT.

Fig 14 illustrates that soft voting performs the best. The dark bar in the figure is the best performing aggregation method. Soft voting outperforms other methods across all r@*k* metrics for both datasets. Although soft voting results with joint probability and averaging are identical, the computational cost of averaging strategy is cheaper. Hard-voting strategies have constant computational complexity.

Despite the promising result of the ensemble model, the practicality of it is limited when it comes to deployment. Deploying such a model would require running multiple models in parallel and then aggregating the results. This is not feasible in real-time applications for the models used in this work. To the best of authors’ knowledge, this is the first work that uses an ensemble model cross-view goe-localization, and it is a promising direction for future work.

### EgoTR fine-tuning

**Fig 15:**
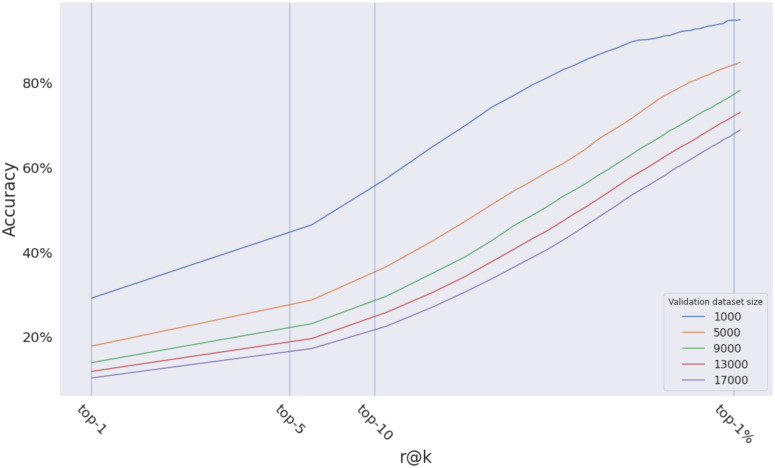
The effect of the size of the validation dataset on the r@*k* metric. Same model (EgoTR) with the same dataset (BDD-trajectories). The accuracy decreases as the size of the validation dataset increases.

The training process took 192 hours for 228 epochs. Table 6 shows the r@*k* metrics for the model. This drop in accuracy can be attributed to: **A)** the ground images in the proposed dataset are not panoramic, in contrast to CVUSA. **B)** high similarity between the consecutive pairs. **C)** one of the shortcomings of the r@*k* metric is that it depends on the size of the validation dataset as shown in Fig 15, the proposed validation dataset size is more than double the size of CVUSA or the size of CVACT.

**Table 6:** r@*k* metrics of EgoTR fine-tuned over the reshaped BDD-trajectories dataset.

r@1	r@5	r@10	r@1%
(%)			
9.99	16.72	21.95	66.58

### Plain naive history

**Fig 16:**
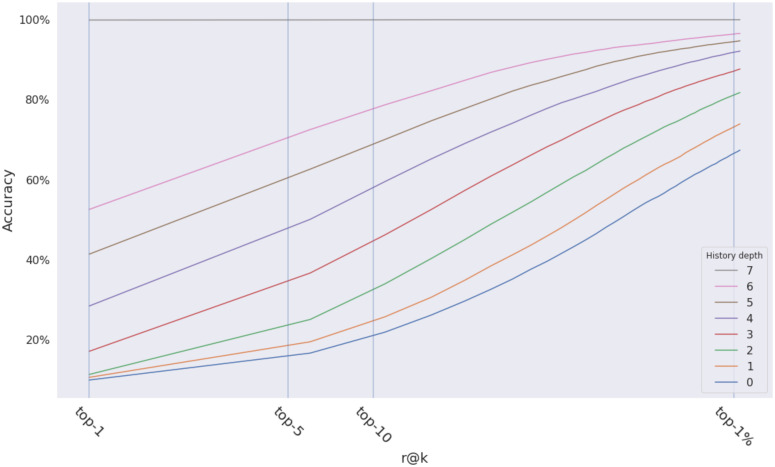
The effect of the number of steps we look back on the accuracy.

The experiments shown in Fig 16, illustrates that the more the naive history algorithm looks back into the history of the journey, the more accuracy improves. The accuracy converges to 100% after seven steps on the proposed dataset. The algorithm has no assumptions about the current state, and its computational cost is negligible compared to generating the distance array.

### Naive history with weak prior

**Fig 17:**
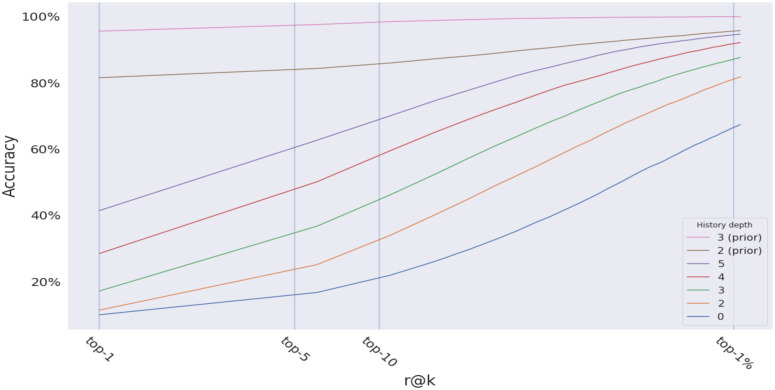
The effect of weak prior on naive history.

Fig 17 shows that naive history with a weak prior with 2 steps look back outperforms plain naive history with 5 steps look back. And it only takes 3 steps for naive history with a prior to converge to 100%, compared to 7 steps (Fig 16) for the plain version. The accuracy of naive history improves significantly when starting with some prior knowledge about the trip starting point. The brown line(naive history with prior) and the green line (plain naive history) in the figure represent the same number of steps into the trip, though the brown line shows more accurate predictions due to factoring in the weak prior.

Despite this promising result, if the prior is wrong it can result in “trapping” the algorithm in the wrong state which will degrade the accuracy significantly, and this is the “naive” aspect of the naive history algorithm.

The “trapping” can be avoided by running the algorithm for a few steps and then running a new instance of the algorithm with a new prior and comparing their predictions. If they diverge, this means one of the instances is trapped. The one that is most faithful to the GPS system should be picked and the other one should get discarded. This can be scaled to a large number of instances by running them in parallel and then comparing their predictions. Compared to the ensemble model, this approach is more practical because it does not require running multiple models in parallel, only the naive history algorithm which consists solely of shifting and multiplication.

The most promising thing about this algorithm is that it can be used in real-time applications and get attached to any cross-view models. For example, it outperforms the GAMa 2D-CNN network [54] which takes 8 steps to get an accuracy of 83% for the r@1% on their proposed dataset which is based on BDD100K too. The proposed algorithm requires minimal resources compared to it during deployment and no training at all.

## Conclusion and future work

In this work, an ensemble model is created to merge the predictions of numerous cutting-edge models. The ensemble model accuracy surpassed the current state-of-the-art. The effect of factoring in trip temporal information is demonstrated. The naive history meta block is proposed, which converges to 100% after few steps. But none of the available benchmark datasets is appropriate for extensive temporal awareness experiments, so a new derivative dataset based on BDD100K is collected. The derivative dataset is used to build an end-to-end model that exploits the temporal correlation during a single trip, and fuses other data modalities and sources during querying and training. It’s evident that there is a room for analyzing different state-of-art models to identify the most promising building modules and then use the network architecture search (NAS) paradigm to develop an optimal CV matching network. The authors anticipate that this study may kick-start the development of deployable cross-view geo-localization models, exploring fusing other data modalities and sources during querying and training. Moreover, the authors believe there is a great gap and need for real-time, weatherproof models, which can initiate several research points.
